# Shape based assignment tests suggest transgressive phenotypes in natural sculpin hybrids (Teleostei, Scorpaeniformes, Cottidae)

**DOI:** 10.1186/1742-9994-2-11

**Published:** 2005-06-29

**Authors:** Arne W Nolte, H David Sheets

**Affiliations:** 1 Institute for Genetics, Evolutionary Genetics, Weyertal 121, 50931 Cologne, Germany; 2 Dept. of Physics, Canisius College, 2001 Main St., Buffalo, NY 14208, USA

## Abstract

**Background:**

Hybridization receives attention because of the potential role that it may play in generating evolutionary novelty. An explanation for the emergence of novel phenotypes is given by transgressive segregation, which, if frequent, would imply an important evolutionary role for hybridization. This process is still rarely studied in natural populations as samples of recent hybrids and their parental populations are needed. Further, the detection of transgressive segregation requires phenotypes that can be easily quantified and analysed. We analyse variability in body shape of divergent populations of European sculpins (*Cottus gobio *complex) as well as natural hybrids among them.

**Results:**

A distance-based method is developed to assign unknown specimens to known groups based on morphometric data. Apparently, body shape represents a highly informative set of characters that parallels the discriminatory power of microsatellite markers in our study system. Populations of sculpins are distinct and "unknown" specimens can be correctly assigned to their source population based on body shape. Recent hybrids are intermediate along the axes separating their parental groups but display additional differentiation that is unique and coupled with the hybrid genetic background.

**Conclusion:**

There is a specific hybrid shape component in natural sculpin hybrids that can be best explained by transgressive segregation. This inference of how hybrids differ from their ancestors provides basic information for future evolutionary studies. Furthermore, our approach may serve to assign candidate specimens to their source populations based on morphometric data and help in the interpretation of population differentiation.

## Background

Although hybridization has long been considered important in the diversification of plants zoologists often considered it detrimental and thus unimportant [1]. The debate of the relative importance of hybridization has received recent attention because the advance of molecular techniques has resulted in a surge of data suggesting that hybridization is taking place rather frequently in the animal kingdom as well. This in turn has revived questions surrounding the potential role that hybridization may play in the penetration of evolutionary novelty in animals [2,3]. A simple explanation for novel phenotypes of hybrids is available through the process of transgressive segregation. Briefly, transgressive segregation is a phenomenon specific to segregating hybrid generations and refers to individuals that exceed parental phenotypic values in any direction. This could be caused by heterosis, which is most pronounced in first generation hybrids, or alternatively by the complementary action of parental alleles dispersed among divergent parental lineages. If this is frequent, then an important evolutionary role for hybridization is more easily explained [4].

In fact, there is abundant evidence that transgressive segregation is common in both plants and animals and that the genetic architecture for it is rather commonplace than exceptional [5]. Given these findings, it is astonishing, that relatively few studies have evaluated transgressive segregation in natural systems [4]. On the one hand this a results from the paucity of study systems where sufficiently large samples are readily available and from the simple fact that quantitative genetics experiments are usually conducted in controlled environments in order to separate environmental from genetic effects. If one searches for transgressive segregation one would ideally study traits, which are determined by several genes and that display a hidden divergence of the underlying genetic network [5]. Finally, one has to study direct hybrids and not lineages of hybrid origin because otherwise secondary evolutionary processes will have reshaped any hybrid lineage and secondarily modified characters cannot be easily distinguished from transgressive traits. Despite these difficulties many evolutionary studies will ultimately have to incorporate natural populations in real ecosystems if the effects and outcome of hybridization are to be analysed.

As an example, hybrid zones among divergent lineages are viewed as natural laboratories and offer interesting study systems [6]. In these, the fitness of hybrids is a key component to understand the dynamics of the hybrid zone as a whole [7]. Transgressive segregation may affect hybrid fitness as it is a mechanism that would make hybrids different and thus produces the raw material upon which selection can act.

We have recently identified hybrid zones of European sculpins belonging to the *Cottus gobio *complex (Scorpaeniformes, Cottidae) that fulfill the above requirements. Sculpins are small, benthic freshwater fishes that occur in streams throughout Europe, with closely related species distributed throughout the northern hemisphere. Previous studies have revealed a high cryptic diversity of this group across the entire distribution range [8]. Our focus area is the River Rhine System, where divergent lineages of sculpins are known to occur in parapatry and have come into secondary contact [8,9]. Small tributaries to the Lower Rhine drainage are inhabited by isolated populations of 'stream' sculpins, a lineage endemic to the River Rhine [8,10]. These stream populations correspond to *Cottus rhenanus *[11]. Intriguingly, a new 'invasive' lineage, has recently appeared within the main channels of larger rivers that where previously free of *Cottus *[10]. The invasive sculpins represent a different species, *Cottus perifretum *[11] that differs from sculpins in streams of the Rhine area (*C. rhenanus*) in body shape and in that its lateral body is largely covered by modified scales vs. an almost complete absence of such modified scales [10]. Invasive sculpins come into secondary contact with populations of stream sculpins where small tributaries disembogue into the main channel of larger rivers. In these areas individuals belonging to both parental populations as well as hybrids among them occur syntopically. With respect to transgressive segregation the above prerequisites are fulfilled. First, sufficiently divergent lineages come into contact and produce recent hybrids. Secondly, these hybrids can be readily identified using genetic data. Finally, variation in body shape provides a well-suited character complex since sophisticated methods are available to study shape [12]. Furthermore, previous studies on body shape show that this character complex is usually determined by multiple genes [13-15]. Below we combine genetic and phenotypic approaches to study body shape in sculpin hybrid zones and present data suggesting that transgressive body shape phenotypes occur in natural sculpin hybrids.

In order to study variation in shape, parental groups and their hybrids were classified to establish how their phenotypes and genotypes were related. Since shape was of key interest, we relied on model based population genetic approaches [16] to independently cluster and assign specimen to their populations of origin or to determine their hybrid status. Such model based clustering is not possible in the analysis of shape because a powerful "theory of population shape" comparable to population genetic theory is lacking that would allow to independently infer population affinity.

However, simple assignment methods can contribute much in the sense of the first assignment approaches in genetics that were employed for much more basic questions [17] namely the problem that distances alone are biologically and conceptually hard to interpret. As an alternative to an abstract distance one may ask whether a given character is sufficiently informative to be diagnostic at the individual, population or at higher levels to help in assessing the significance of results. This general problem also applies to quantitative morphometric studies, especially when multivariate analyses are used. We have developed a distance based assignment approach with statistical tests that parallel population genetic approaches [18]. The method is not intended to give a measure of the absolute distance among groups but may help to interpret the differences among groups. One purpose of this paper is to introduce shape based assignment as a multivariate measure of distinctness and to employ this approach to study the relationships of genotypes and phenotypes at natural hybrid zones.

## Results

### Morphometric differentiation

**Figure 1:**
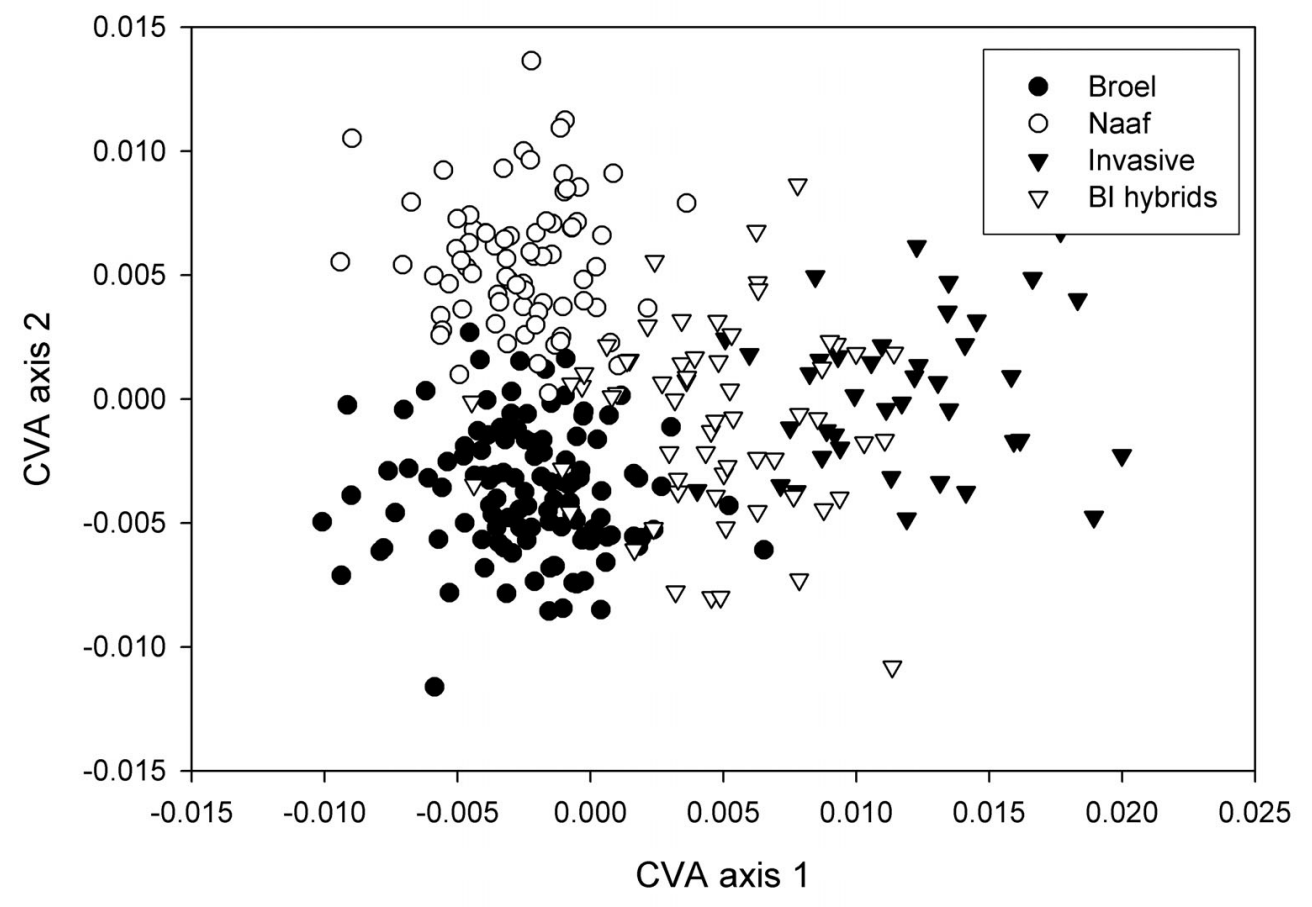
Differentiation of ancestral populations and hybrid intermediacy. Invasive sculpins separate from all stream sculpins along the first CV axis. Sculpin populations from Stream Broel and Stream Naaf separate along the second CV axis. BI hybrids form an intermediate group between their parental populations. Distance based assignment based on these two axes correctly identifies pure candidates while a majority of BI hybrids are wrongly assigned to one of the parental groups with which they overlap.

One population of invasive sculpins (*C. perifretum*) and the two populations of stream sculpins (*C. rhenanus*) each confined to a separate stream were sampled and independently confirmed with genotypic data (see methods; see Additional file: 1). These served as basic groups for the following analyses. All of them form distinct clusters in a canonical variates analysis (CVA) along the first two axes, which display the greatest separation of the groups relative to within group variance (Figure 1). Both populations of stream sculpins separate from the invasive sculpins along the first CV axis (Lambda = 0.0678 chisq. = 777.6114 df = 72 p < 0.001). The stream Naaf and the stream Broel populations are further separated along the second CV axis (Lambda = 0.2792 chisq. = 368.6997 df = 46 p < 0.001).

**Table 1:** Assignment success under alternative CVA models

Assigned Group	Broel	Naaf	Invasive	BI Hybrids	CVA model
Broel	**90.6**	1.3	2.5	25.8	Based on parental populations
Naaf	4.3	**93.4**	0.0	11.3	
Invasive	0.9	0.0	**92.5**	25.8	
n.s.	4.3	5.3	5.0	**37.1**	
Broel	**87.2**	5.3	0.0	1.6	Including parental groups and hybrids
Naaf	2.6	**89.5**	0.0	1.6	
Invasive	0.9	0.0	**85.4**	6.5	
BI hybrid	6.8	1.3	12.2	**83.9**	
n.s.	2.6	3.9	0.0	6.5	

Assignment of sculpins to their population of origin based on body shape. A model based only on the differentiation of parental populations is very effective in identifying pure sculpins but assigns the majority of hybrids to pure populations as false positives. A more complex model that includes the shape components specific to hybrids correctly identifies the majority of all hybrids. The overall success of parental group assignment is decreased when hybrids are taken into account as they overlap with parental phenotypic values.

**Figure 2:**
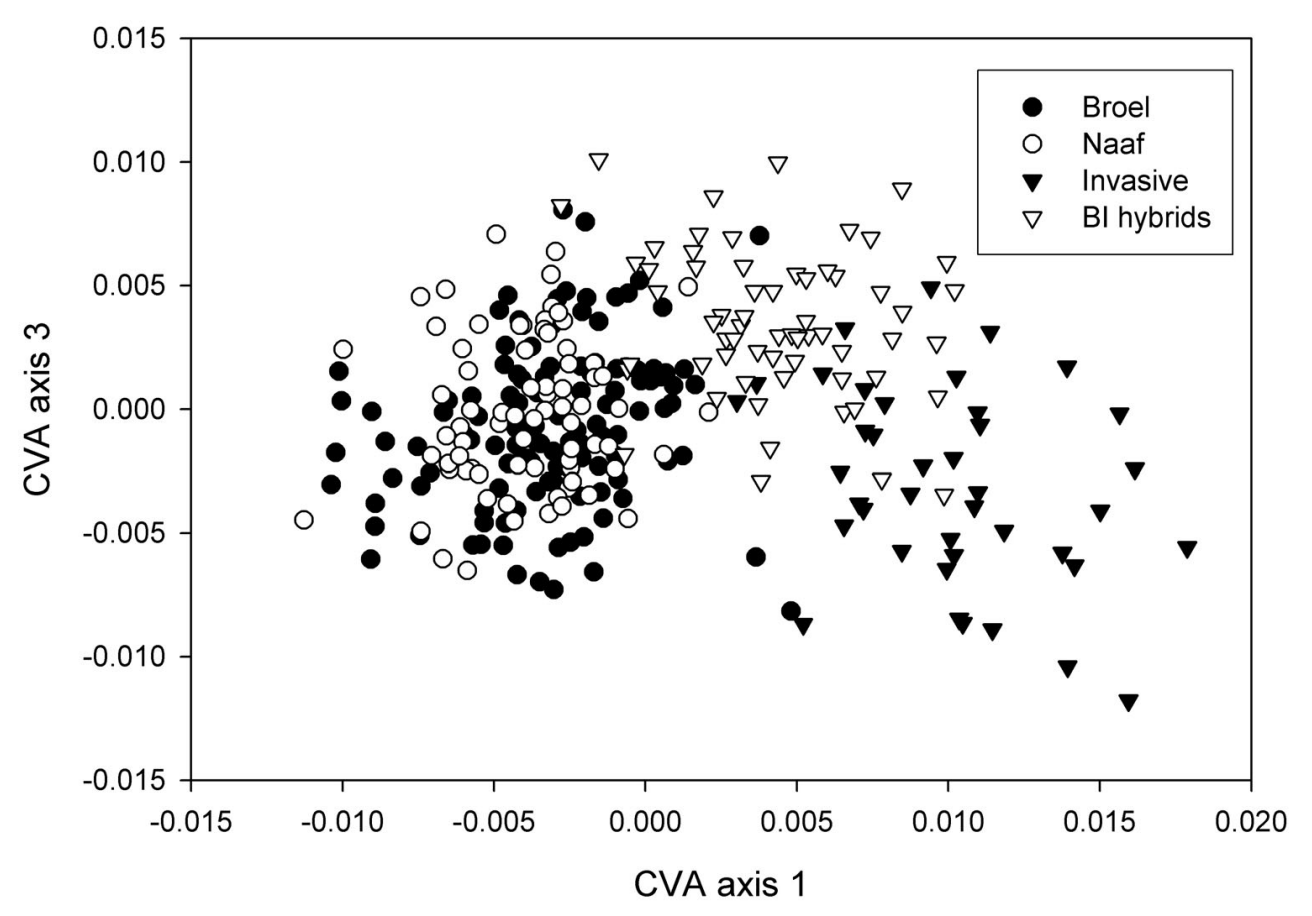
Extreme phenotypic values indicate a hybrid shape component. BI Hybrids are, on average, not intermediate along the third CV axis and may occupy extreme values relative to their parental populations. The parental populations as well as stream Naaf sculpins display little differentiation along the third CV axis. An inclusion of this hybrid specific shape component in distance based assignment increases the power to correctly identify hybrids more than two fold.

A fourth group comprised recent hybrids among invasive and stream Broel sculpins that were sampled from natural hybrid zones and identified based on genetic data (BI hybrids). When these are introduced into the CVA as 'unknowns', where the CVA model does not consider them as a separate group but determines their scores along the CV axes separating the parental groups, BI hybrids overlap with their ancestral populations and take somewhat intermediate positions along the first CV axis (Figure 1). If in contrast BI hybrids are used as a predefined group in the CVA, hybrids are further characterized by a third CV axis (Lambda = 0.7369 chisq. = 88.2487 df = 22 p < 0.001). They separate partially from invasive sculpins and stream sculpins along the third CV axis (Figure 2) and take, on average, more extreme phenotypic values than both parental populations along this axis.

**Figure 3:**
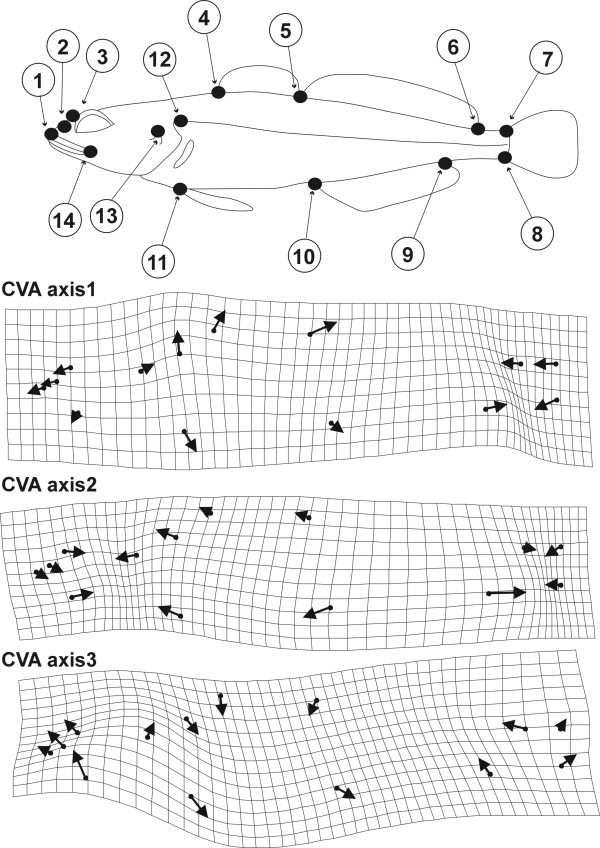
Landmark configuration and displacement vectors that distinguish groups of sculpins. Fourteen Landmarks were chosen to analyse variability in sculpin body shape (top). CVA was used to identify axes along which different groups can be discriminated based on the relative position of landmarks to a reference. The shape change captured by these axes can be visualized as relative displacement vectors for each landmark on a deformation grid. CV axis 1 separates invasive sculpins from all stream sculpins and axis 2 further separates two populations of stream sculpins. CV axis 3 captures the shape component that is unique to recent hybrids. While the deformation along CV axes 1 and 2 can be expressed in terms of inflation or compression of body parts, the hybrid specific shape change appears to be less balanced.

The differentiation in shape as captured by CV axes can be visualized as displacement vector for each landmark on a deformation grid relative to a reference (Figure 3). Invasive sculpins differ from both populations of stream sculpins in that they have a larger head and anterior trunk as well as a shorter tail (Figure 3; first CV axis). The two populations of stream sculpins differ most in their head length and the positions of their anal and dorsal fin landmarks (Figure 3; second CV axis). While the deformation implied by the first two axes can be expressed in terms of inflation or compression of body parts, the hybrid specific shape change appears to be less balanced although this is hard to objectify (Figure 3; third CV axis).

In order to evaluate whether the observed differentiation was biased due to imbalanced sampling CVA scores were regressed on centroid size and sex for all specimens. A linear regression reveals for all axes that correlations coefficients were low at most (CV axis1 vs. size r^2 ^= 0.03; CV axis1 vs. sex r^2^< 0.01; CV axis^2 ^vs. size r^2^<0.01; CV axis2 vs. sex r^2^<0.01; CV axis3 vs. size r^2 ^= 0.11; CV axis3 vs. sex r^2 ^= 0.019). Therefore, neither size nor gender can explain the considerable amount of variance of the CV axes that distinguish the groups.

### Assignment and cross validation

To evaluate the utility of the derived axes to discriminate among groups and to determine a given specimens group affinity, distance based assignment tests were performed. In a first approach the CVA model was based on the differentiation as observed among the pure populations but hybrids were not included as a known group. The clear differentiation of pure populations facilitates that single "unknown" specimens removed from the complete dataset can be correctly assigned to their population of origin with high success (Table 1), and the resubstitution rate of correct assignment (the assignment of the known specimens) is high also, although this resubstitution rate is known to be biased upward. Approx. 92 % of pure sculpins were assigned correctly. This number is slightly lower than the expected 95% due to false positive assignments because of a slight overlap of parental phenotypic values. The number of outliers corresponds well to the amount expected from the significance criterion. In this approach hybrids were used as "unknowns" and could only be identified as outliers relative to the pure sculpins (non significant assignment test). Only 37.1% of the BI hybrids were correctly classified while the majority was misassigned to one of the pure populations.

In an alternative approach assignment was based on a CVA model that includes the differentiation among pure populations but also takes into account the shape component specific to hybrids as captured by the third CV axis (Figures 2 &3). The assignment success of pure populations was decreased to 85.4–89.5% because of the partial overlap with the group of hybrids. In sharp contrast to the above, 83.9% of the BI hybrids were now classified correctly with only relatively few false positive assignments to the parental groups (Table 1).

A jackknife test of assignment was performed for both assignment approaches to evaluate the robustness of the CV axes and assignment model (Table 2). The cross validation procedure revealed a very consistent signal, inherent to even small partitions of the whole dataset. Roughly half of the specimens can be removed from the data without much loss of information for the CV axes. Even when 80% of the whole dataset are left out in the CVA procedure (see methods) the general outcome remains unchanged although the number of correct assignments decreases. As evident from the individual assignment tests (Table 1) the overall assignment success is lower when the more complex model including hybrids is employed.

**Table 2:** Jackknife estimates of assignment performance.

% left out in Jack – knife (500 replicates)	1	10	50	80	CVA model
% correct	**93.6**	**92,7**	**90.3**	**72.1**	Based on parental groups
% correct ns.	0.5	0.5	1.3	10.5	
% false	5.9	6.8	8.3	14.8	
% false ns.	0.0	0.0	0.1	2.6	
% correct	**84.7**	**84.3**	**80.4**	**65.3**	Including parental groups and hybrids
% correct ns.	0.1	0.0	0.4	4.2	
% false	15.3	15.6	19.2	28.9	
% false ns.	0.0	0.0	0.1	1.6	

Jackknife test of assignment. Percent correct and false assignments when fractions of 1% to 80% of the specimens are left out in the CVA procedure and then assigned to groups in the remaining dataset. Large fractions of the data can be removed without loss of the discriminatory power of CVA axes.

## Discussion

### Transgressive phenotypes in natural hybrids

We were able to use a microsatellite dataset with surpassing information content to classify sculpins into distinct lineages (see methods for details). With the genotypic data it is possible to unambiguously identify invasive sculpins (*Cottus perifretum*), which are genetically distinct from populations of stream sculpins (*Cottus rhenanus*). The latter are further represented here by two separate populations from the streams Naaf and Broel. In agreement with the genetic data, the CVA based on morphometric data recovers significant differentiation that separates all studied populations of sculpins with a higher amount of variance between species and a lesser amount between two conspecific samples ofstream sculpins. Cross validation confirms that these results are based on a signal inherent to the whole dataset as a removal of a large fraction of the specimens will not notably alter the axes as determined in the CVA (Table 2). This differentiation is sufficient to assign unknowns to either one of the known groups with high confidence. Thus the groups are distinctly different in their multivariate signal even though no single diagnostic morphometric character can be found.

The genotypic data served to identify a set of hybrids between the invasive and stream Broel populations (BI hybrids). In contrast to the ancestral populations, hybrids cannot be distinguished completely from all of the ancestral phenotypes (Figure 1) and are more or less intermediate in the characters that discriminate their parental populations. This is expected for a character like body shape that is most likely determined by multiple genes. Yet, there are properties of the hybrids that could not be attributed to hybrid intermediacy. The group of hybrids displays a unique shape component that distinguishes them from a their parental populations (Figure 2). Altogether it is not a strong effect thus additional evidence to evaluate the biological significance of this result are desirable. To address sampling artifacts, we have tested for possible effects of typical confounding variables in morphometric studies. Regressions show that the amount of total variance of individual CV axis scores that can be explained by size or sex is small. Therefore the influence of allometry or sexual dimorphism is most likely not important for the differentiation we observe. Despite the large overlap of the BI hybrids with the parental populations, the hybrid shape component constitutes a considerable amount of variation, which results in an increased overall assignment success when hybrid shape is considered specifically (Table 1). Moreover, cross validation has shown that all axes are robust to removal of specimens, which suggests that the signal is inherent to a majority of the recent hybrids (Table 2).

Two alternative explanations remain. One assumes an involvement of genetic factors that interact to produce novel phenotypes, in contrast, the second proposes that the genetic background is not important. According to the latter hypothesis extreme hybrid phenotypes should be determined by the environment. Our genetic data demonstrate that hybrids occur syntopically with the parental populations within the hybrid zones (Table 3). This excludes the possibility that hybrids would be exclusively subjected to environmental factors that could induce the observed phenotypes. Phenotypic plasticity cannot be fully excluded in heterogeneous environments but this process alone is not likely to explain our results. After possible confounding variables were found to play a minor role, it seems reasonable to assume the differentiation is real. In contrast to the above explanations, differentiation due to the underlying genetic background is strongly supported. This includes that the specific hybrid shape effect is coupled with the hybrid genetic background.

**Table 3:** Sampling sites and number of specimens in the morphometric study.

**Number**	**Sampling Site**	**N (Genotyped)**	**N (Genotypic Classes)**
1	Stream Broel between Broel and Winterscheidt, North Rhine-Westphalia, Germany; 50°47'N 7°20'E	48	48 Stream Broel sculpins
2	Stream Broel south of Broel, North Rhine-Westphalia, Germany; 50°47'N 7°19'E	48	42 Stream Broel sculpins; 1 BI Hybrid
3	Stream Broel at Mueschmuehle, 200 m above outlet into River Sieg, North Rhine-Westphalia, Germany; 50°47'N 7°18'E	130	26 Stream Broel Sculpins; 45 BI Hybrids; 13 Invasive Sculpins
4	River Sieg at Allner, below outlet of Stream Bröl, North Rhine-Westphalia, Germany; 50°46'N 7°18'E	36	2 Stream Broel Sculpins; 16 BI Hybrids; 2 Invasive Sculpin
5	Stream Wahnbach, Outlet into River Sieg at Seligenthal, North Rhine-Westphalia, Germany; 50°47'N 7°16'E	4	1 Invasive sculpin
6	Stream Pleis, outlet into the River Sieg at Niederpleis, North Rhine-Westphalia, Germany; 50°46'N 7°12'E	5	5 Invasive sculpin
7	River Sieg at Muehlenbach, North Rhine-Westphalia, Germany; 50°47'N 7°10'E	35	19 Invasive sculpin
8	Stream Naaf, Outlet into River Agger, North Rhine-Westphalia, Germany; 50°51'N 7°14'E	48	1 Stream Naaf sculpins;
9	Stream Naaf at Kreuznaaf, 200 m above outlet into River Agger, North Rhine-Westphalia, Germany; 50°51'N 7°14'E	48	30 Stream Naaf sculpins
10	Stream Naaf southeast of Hausdorp, North Rhine-Westphalia, Germany; 50°52'N 7°16'E	48	45 Stream Naaf sculpins

Sampling Sites, total number of genotyped specimens and numbers in genotypic classes used for this study. The individual genotypic classes were inferred from microsatellite data and served to group specimens for morphometric analyses. Specimens excluded from the analysis include later generation backcrosses or those for which morphometric data could not be obtained. Note that there are sampling sites at which all genotypic classes occur syntopically.

Although there is considerable overlap of parental groups and BI hybrids along the CV axis that captures the hybrid shape component, hybrids are on average more extreme than both parental populations. Given that genetic data verify a recent hybrid status of the BI hybrids, these results can be best explained by transgressive segregation in shape traits. This is also in agreement with other studies suggesting that transgressive segregation occurs in morphometric traits [4,5]. However, to assess the evolutionary implications of the hybrid phenotypes will require functional studies and measurements of fitness to complement the mere observation of possible transgressive effects.

### Information content of shape markers

A drawback as compared to population genetic model – based assignment is that our shape distance based method needs a priori defined groups as input. If such groups can be provided hypotheses regarding their differentiation and distinctness can be tested. For example, an attractive application of genotype based assignment procedures is to detect outliers that belong to source populations that were not sampled [19,16]. Unfortunately this is not straightforward in our implementation of phenotype-based assignment. If a candidate does not belong to one of the expected groups, the exclusion of source populations is not predictable because assignment based on discriminant axes is conditioned on the specific case being studied. We find this for the hybrids among stream Broel and Invasive sculpins if they are used as unknowns and not as a separate group in the CVA. Hybrids take more or less intermediate phenotypic values but largely overlap with the parental groups (Figure 1).

Similar results were already obtained by Strauss [20] in a study of phenotypic variation in hybridising North American sculpins. However, we have a sufficiently large sample of verified hybrids that could be used as an extra group in the CVA. Only this revealed significant differentiation along an extra axis that is specific to the hybrids. The differentiation specific to hybrids adds information to the group assignment. As a result the assignment success of hybrid specimen was raised notably despite the tremendous overlap of the hybrids with both parental populations (Table 1).

The assignment procedure based on morphometric data as implemented here allows to unambiguously assign sculpins to their population of origin. Morphometric differentiation of European sculpins was studied before [21] using a set of landmarks that was largely identical to the ones used here (note that these authors [21] did not study the same evolutionary lineages, see [8,11]). Groups of sculpins as defined by different tributaries to the Rhine were found to differ significantly in shape but formed largely overlapping clusters. Our system differs in that we have not compared assemblages of populations but separate more or less panmictic populations as defined by a currently shared gene pool. These form distinct clusters in the CVA (Figure 1). Such differentiation would have escaped the approach of Riffel and Schreiber [21] as they pooled specimens from different subpopulations for their analysis. This by no means negates their results but demonstrates an even higher information content of shape data, namely the power to discriminate separate populations. Although a comparison among genetic markers and body shape seems arbitrary the resolution as compared population genetic markers goes beyond recognition of ancient lineages as resolved by mitochondrial haplotypes [8] and species [11] but parallels that of microsatellites in that genetically well separated populations are also distinctly differentiated in body shape. Apparently, shape represents a character with a fast evolutionary divergence that occurs and becomes fixed even among closely related populations. Thus, in our example morphometric data resolve to the lowest possible level above the individual.

## Methods

### Implementation of shape based assignment

Landmark-based geometric morphometric methods were used to capture information about shape, by obtaining the x and y coordinates of homologous landmarks in the configuration shown in Figure 3. Differences among specimen in the sets of coordinates due to scaling, rotation and translation were removed using the typical geometric morphometric approach [22-25,12] of placing the specimens in Partial Procrustes Superimposition [24-26] on the iteratively estimated mean reference form, using the Generalized Procrustes Analysis procedure. This procedure places the shapes of specimens in a linear tangent space to Kendall's shape space [27], allowing the use of linear multivariate statistical methods [23,28,24,12]. After superimposition, the data were converted from Cartesian coordinate form into components along the eigenvectors of the bending energy matrix (Principal Warp axes) of the thin-plate spline model of deformations of the reference [29,22] and along the uniform axes of deformation due to shear and dilation [30]. Use of these linearly transformed variables (referred to as Partial Warp plus Uniform Component scores), produces a convenient set of variables (using a basis set called the Principal Warp axes) for use with standard multivariate statistical methods, since the Partial Warp and Uniform component scores have the same number of variables per specimen as degrees of freedom. No information is lost during this linear transformation of variables.

A canonical variates analysis (CVA) is then used to determine the set of axes which best discrimate among pre-defined groups of specimens, by determining the linear combinations of the original variables which display the greatest variance between groups relative to the variance within the groups [31,12]. Fisher's linear discriminant function was used, which makes no particular assumption about the parametric form of the distribution of the data used, but simply determines the linear combination of the original variables that results in the greatest ratio of the between groups sum of squares to the within groups sum of squares [32]. A simple distance-based approach is then used to determine which group each specimen belongs to, based on the canonical variate scores. The predicted group membership of each specimen based on the CVA scores is determined by assigning each specimen to the group whose mean is closest (measured as the square root of summed squared distances along the CV axes, see [32]) to the specimen. To obtain a measure of the quality of the assignment of each specimen to a group, an assignment test was developed. The CVA axes can always be used to assign any given specimen to some group, since a minimum distance can always be found but a measure of whether the quality of the assignment is similar to that expected for specimens known to be in that particular group is desirable. The assignment test presented here is modeled on the genetic distance-based assignment test [33,18]. The distribution of distances produced by a Monte Carlo simulation (see discussion in [34]) is used to determine if the observed distance of a given specimen is consistent with the null model of random variation around the mean of the group to which the specimen is assigned to. The distance from a specimen to a group mean can then be assigned a p-score which describes how likely it would be for a specimen from the original population to be as far from the mean specimen as the observed specimen is (under the null model used in the Monte-Carlo simulation). If the p score is smaller than 5%, then we can assert that there is a less than 5% chance that random variation could have produced a distance as large as that of the particular specimen from the group mean, and hence that the assignment of that specimen to the group is in doubt.

It should be noted that in a study with many specimens, a number of them will have low p-scores by chance, and so to assess the validity of the assignments of the set as a whole, the researcher should assess the number of specimens expected to have p values less than 5%. It will then be possible to determine if the observed number of low p values exceeds that expected by chance. The model used in the Monte Carlo simulation of the distribution of distances of specimens within a group around the group mean (the average specimen within the group) is based on a normal model of the distribution of the CVA scores of each group about the mean of that group. For a given group, it appears probable that the CVA scores along each CVA axes for the specimens within the group are correlated, thus there exists within each group a covariance structure to the CVA scores of specimens within the group. In carrying out the Monte-Carlo simulation of the distribution of specimens within the group about the mean, it is necessary to preserve this covariance structure in order to produce a valid model of the distribution. An eigenvalue decomposition of the variance-covariance matrix of within group CVA scores is used to find the principal component axes of the within group variance. This yields the same number of variables as the CVA scores, but now with uncorrelated axes (the eigenvectors), each of which has a variance given by the corresponding eigenvalue. The model used for the distribution of the CVA scores of the specimens assumes the group has an independent random normal distribution along each of these eigenvectors (principle component axes), with amplitudes given by the square roots of the eigenvalues (the eigenvalues are the variances of the group along the corresponding eigenvectors), so that the square root of the eigenvalue is the standard deviation of the population along that eigenvector.

An independent, normal distribution with known amplitude (the square root of the eigenvalue) is assumed along each eigenvector. This allows generation of a Monte Carlo population of specimens, assuming the independent normal distribution along these principal component axes. Each simulated specimen is generated using a random number generator to compute locations along the eigenvectors, which are then translated back into CVA axes scores (a simple linear translation of basis vectors). The Monte Carlo generated CVA axis scores will have the same mean and variance-covariance structure as the original population did. Using an independent multinomial distribution model of the CVA axes scores in the Monte-Carlo simulation (by using the random number generater to directly generate the CVA scores) would not preserve any covariation structure in the data. If there is no significant covariation structure to the CVA scores, the use of the PCA axes is not necessary, but will not induce covariations.

The distance from the group mean is then calculated for each simulated specimen. The Monte Carlo distribution of distances of specimens about the group mean under the null model of random variation can then be used as an estimate of the distribution of distances about the group mean in the original data. Based on the estimated distributions of distances about the group means expected for specimens in the group, p-values may be determined for the assignment of specimens, with either initially known or unknown group affinities. Based on an alpha level of p = 0.05, all assignments of specimens to groups can be scored as either statistically significant or not.

As a test of the performance of the assignment test, a cross validation or jackknife procedure [34,35] was implemented. Unlike a standard jackknife where only one specimen at a time is omitted, a "delete-d" jackknife [35] was used in which d specimens at a time were omitted. Under the delete-d jackknife, a variable percentage of individuals from a dataset are left out during the CVA procedure, and then assigned to groups as "unknown" specimens. The specimens treated as unknowns during the jackknife procedure are also assigned an assignment p-value during this procedure. High success rates under the delete-d jackknife resampling indicate that the differentiation among the involved groups is sufficient to be diagnostic. This implies that the discriminant axes capture enough information to assign individuals of the given groups, and form a reasonable estimate of the distribution of distances based on the Monte Carlo procedure. The jackknife procedure also allows estimation of the number of individuals needed to obtain meaningful CVA axes, and distance distribution estimates.

### The sculpin data set

Here we employ the methods outlined above to study the differentiation in shape that occurs among divergent populations and their naturally occurring hybrids. Population affinity and hybrid status are independently derived from genetic markers. Note that the specimen are taken from natural populations and occur syntopically in the studied hybrid zones (Table 3). Sculpins were sampled across an area of secondary contact of invasive and stream sculpins (*C. perifretum *and *C. rhenanus*) in the Lower Rhine basin, which is situated at the confluence of the Stream Broel with the River Sieg (Table 3). An extra population of stream sculpins was sampled from the stream Naaf (also a tributary to the River Sieg drainage).

All specimens were genotyped for 45 microsatellite loci [36]. The loci were chosen for their particularly high information content for our study system following the approach of Shriver et al. [37] using Whichloci [38]. We have used a preliminary genetic map of *Cottus *[39], to verify that our set of microsatellite markers does not contain pairs of loci that are tightly physically linked. The genotypic data allow to unambiguously classify individuals to belong to pure populations or to identify them as hybrids with a mixed ancestry using the methods outlined in Falush et al. [16]. The program Structure 2.1 [40] yielded consistent results in independent runs (burnin: 20000; sampling iterations 100000, correlated allele frequency model allowing for an individual alpha and different F_ST _for each subpopulation) according to which the genetic ancestry of individual could be determined (see Additional file: 1, for genotypic data of those specimen included here). The classification based on genotypic data was highly congruent with data from distribution and morphology.

Two independent populations of stream sculpins confined to separate streams (Stream Naaf, Stream Broel) both being devoid of skin prickling were recovered. A third population can be identified, which represents the invasive sculpin. Invasive sculpins generally occur within the main channel of the River Sieg and display pronounced skin prickling [10]. Hybrids among Stream Broel sculpins with the invasive sculpins were only found at the confluence where the Broel merges with the River Sieg (Table 3). A detailed study of these hybrid zones, particularly on the geographic extension, is currently in preparation (Nolte *et al. *in prep). Of particular relevance in this context is the fact that hybrid sculpins occur syntopically with their parental populations within the hybrid ones (Table 3; Sites 2, 3, 4). For the morphometric analysis we grouped specimens that were found to belong to pure populations from the genotypic data into those corresponding to the invasive sculpins (invasive) and to the two stream sculpin populations (Streams Naaf and Broel). To allow for some uncertainty in the estimates we used those specimens that were found to be at least 97% pure in the structure analysis. These populations are represented here by 117 Stream Broel sculpins, 76 Stream Naaf sculpins and 40 Invasive sculpins (Table 3). In contrast, hybrids represent a somewhat inhomogeneous group consisting of various degrees of ancestry. To restrict this analysis to those specimens that have a pronounced hybrid genotype and to exclude later generation backcrosses that might have been subject to repeated rounds of natural selection (as this could blur possible transgressive effects) we decided to exclude hybrids with less than 25% ancestry in one of the ancestral populations. Based on genotypic data, we were able to identify 62 BI hybrids (mixed Stream Broel/Invasive ancestries, less than 75% pure ancestry).

Images of specimens were taken with a digital camera fixed on a stage so that the midsagittal body plane was as much as possible perpendicular to the image plane. Fourteen morphological landmarks were used to capture the shape of each individual (suppl. Table 2) using TPSdig [41]. The positions of the tip of the nasal (#1), nares (#2), interorbital pores (#3), dorsal fin I origin (#4), dorsal fin II origin (#5), dorsal fin II end (#6), upper caudal fin origin (#7), lower caudal fin origin (#8), anal fin end (#9), anal fin origin (#10), ventral fin origin (#11), upper origin of the gill opening (#12), opercular spine (#13) and posterior end of the maxilla (#14) were used as landmarks (Figure 3). The morphometric analysis was conducted using the IMP package according to the methods outlined above [42]. Shape based assignment tests were conducted with CVAgen6N (part of IMP). In order to estimate possible confounding effects of allometric growth and sexual dimorphism these variables were determined individually. A scale bar was photographed besides each specimen as a size reference and sex was determined for individuals larger than 45 mm by examination of the genital papilla (see Additional file: 2).

## Authors' contributions

AN identified the sculpin hybrid zones and carried out the molecular genetic studies, morphometric analyses and drafted the manuscript. HDS participated in the morphometric analysis, developed the assignment procedure based on morphometric data and helped to draft the manuscript. All authors read and approved the final manuscript.

## Supplementary Material

Additional File 1 Inferred group affinity and individual genotypic data. Genotypes of all specimens for 45 microsatellite loci (0 = missing data, alleles numbered according to size, but not necessarily repeat size) with group affinity and sampling site as of Table 3. Click here for file

Additional File 2 Individual landmark data, centroid size and sex. Cartesian coordinates (X – Y format) for fourteen landmarks, with individual group affinities and sampling site as of Table 3 as well as sex (0 = female; 1 = male) and centroid size. Click here for file
